# Antcin K suppresses proinflammatory cytokines expression via the PI3K, Akt and NF-κB pathways in human gingival fibroblasts: implications for periodontitis treatment

**DOI:** 10.1038/s41420-025-02865-3

**Published:** 2025-11-22

**Authors:** Ya-Hsin Wu, Yueh-Hsiung Kuo, Yen-You Lin, Tzong-Ming Shieh, Tzu-Ching Chang, An-Chen Chang, Ju-Fang Liu, Chih-Hsin Tang

**Affiliations:** 1 School of Dentistry, China Medical University, Taichung, Taiwan; 2 Department of Periodontology, China Medical University Hospital, Taichung, Taiwan; 3 Department of Chinese Pharmaceutical Sciences and Chinese Medicine Resources, China Medical University, Taichung, Taiwan; 4 Chinese Medicine Research Center, China Medical University, Taichung, Taiwan; 5 Translational Medicine Center, Shin Kong Wu Ho-Su Memorial Hospital, Taipei, Taiwan; 6 Department of Pharmacology, School of Medicine, China Medical University, Taichung, Taiwan; 7 School of Oral Hygiene, College of Oral Medicine, Taipei Medical University, Taipei City, Taiwan; 8 Department of Medical Laboratory Science and Biotechnology, College of Medical and Health Science, Asia University, Taichung, Taiwan

**Keywords:** Cell death and immune response, Expression systems

## Abstract

Numerous inflammatory cytokines control the pathogenesis of periodontitis, an infectious bacterial disease, via interacting with immune and tissue cells. *Antrodia cinnamomea* is the origin of the triterpenoid Antcin K, renowned for its immunomodulatory and anti-inflammatory properties. However, the therapeutic performances of Antcin K on periodontitis remain unclear. Lipopolysaccharide (LPS) is the primary virulence factor of *Porphyromonas gingivalis*, a common periodontal pathogen, which augments the synthesis of proinflammatory cytokines for instance IL-1β, IL-6, IL-8, and IL-17A in primary human gingival fibroblasts (HGFs). Interestingly, treatment of HGFs with Antcin K inhibited LPS-induced proinflammatory cytokines production. RNA sequencing analysis indicated that the PI3K-Akt pathway is potentially linked in Antcin K’s anti-inflammatory function. We revealed that the PI3K, Akt, and NF-κB pathways mediate Antcin K’s suppression of proinflammatory cytokines production. Specifically, our in vivo study demonstrated that Antcin K blocks pathogenesis of periodontal disease in a ligature-mediated periodontitis model. Therefore, we suggest that Antcin K may be a potential therapeutic candidate for controlling periodontal disease.

## Introduction

Periodontitis is a well-recognized chronic inflammatory disease that affects individuals globally, characterized by gradual destruction of the periodontium, including periodontal ligament and alveolar bone [1]. While microbial dental plaque is the primary initiating factor, the severity and pattern of periodontal tissue destruction largely depend on the host’s immune response to bacterial stimulation. When excessive activation of the host immune response occurs, it leads to chronic inflammation and irreversible alveolar bone absorption [2]. If left untreated, this process results in the destruction of connective tissue attachment and eventual tooth loss [3]. *Porphyromonas gingivalis* (*P. gingivalis*), a Gram-negative anaerobic bacterium, is the most crucial periodontal pathogen regulating to the development of periodontal disorders. *P. gingivalis* secretes the bacterial endotoxin lipopolysaccharide (LPS), which enhances bone resorption and triggers an inflammatory response by activating Toll-like receptor 4, thereby accelerating the onset of periodontitis [4, 5].

The most prevalent cells in gingival connective tissues are human gingival fibroblasts (HGFs) [2, 6]. In addition to passively reacting to oral bacteria that breach the epithelial barrier and regulate to inflamed gingival tissue, there is strong data from in vitro studies that HGFs can adopt induced proinflammatory activities that promote the growth of inflammophilic pathogens and regulate the chronicity of inflammation [3, 7]. Gingival tissue health is dependent on HGFs’ fundamental roles in regeneration and repair. Furthermore, HGFs aid in the regulation of inflammatory cascades in periodontal diseases [8]. HGFs responses to increased inflammatory cytokines, participate to the progression of periodontitis since the condition is featured by an imbalance in the metabolism of collagen [9].

As part of a recent trend in the discovery of anti-inflammatory medicines, investigators are actively searching for active anti-inflammatory ingredients in natural pharmaceuticals and investigating the mechanisms underlying their anti-inflammatory effect [10–12]. Inflammatory illnesses now have a novel therapeutic alternative in the form of natural substances [13, 14]. Known for its strong anti-inflammatory, hepatoprotective, anti-cancer, immunomodulatory, and anti-oxidative properties, *Antrodia cinnamomea* (*A. cinnamonomea*) is a rare medicinal fungus that is indigenous to Taiwan [15–17]. In vitro and in vivo investigations have documented the anti-angiogenesis and anti-inflammatory properties of Antcin K, a triterpenoid isolated from *A. cinnamonomea* [18, 19]. In chondrosarcoma, Antcin K inhibits metastasis by suppressing MMP-7 production [20]. Specifically, Antcin K displayed potential anti-arthritic activity due to its anti-inflammatory abilities [19, 21]. A recent report indicates that Antcin K reduces skeletal muscle injury and inflammation by enhancing IL-10 production [22]. Nevertheless, the potential anti-inflammatory performances of Antcin K in HGFs and its role in periodontitis treatment remain unclear. Therefore, this study explores the promising therapeutic potential of Antcin K, investigating its anti-inflammatory properties and protective effects against alveolar bone loss in periodontitis, with the goal of elucidating the molecular mechanisms that could establish Antcin K as a novel therapeutic agent for periodontal disease management.

## Results

### Antcin K inhibits proinflammatory cytokines production in HGFs

**Fig. 1:**
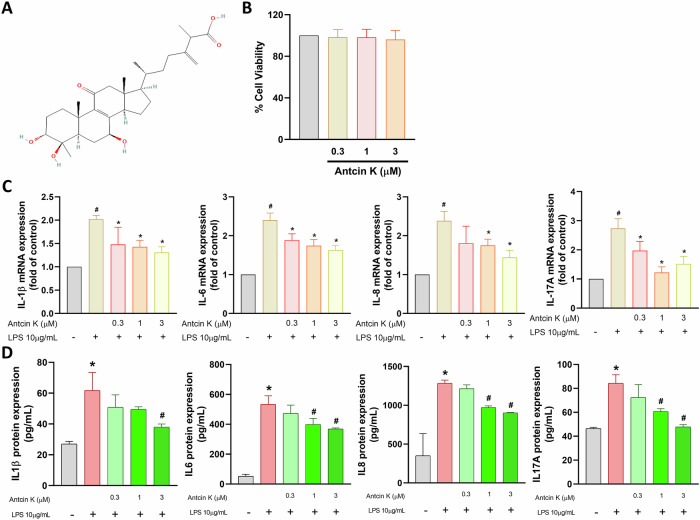
Antcin K inhibits proinflammatory cytokines production in HGFs. **A** Chemical structure of Antcin K. **B** HGFs were treated with Antcin K (0.3–3 μM) for 24 h, the cell viability was performed by MTT assay. **C**, **D** HGFs were stimulated with LPS and Antcin K for 24 h, the mRNAs and protein synthesis was analysed by qPCR and ELISA assay. *p < 0.05 compared with the control group. ^#^*p* < 0.05 compared with the LPS-stimulated group.

A chronic inflammatory disorder that affects the tissues that surround and support teeth is named periodontal disease [1]. In this study, we used primary HGFs, the most prevalent cells in gingival connective tissues, to examine the anti-inflammatory properties of Antcin K [8]. According to the MTT assay, Antcin K did not affect cell viability (Fig. 1A, B). LPS stimulation in HGFs increased the synthesis of proinflammatory cytokine mRNAs, including IL-1β, IL-6, IL-8, and IL-17A, as well as their protein expression in the culture medium (Fig. 1C, D). Antcin K treatment markedly inhibited LPS-induced production of these proinflammatory cytokines concentration-dependently (Fig. 1C, D). Therefore, Antcin K effectively blocks the proinflammatory response in periodontal disease.

### The suppressive effects of Antcin K are mediated through the PI3K, Akt, and NF-κB signaling pathways

**Fig. 2:**
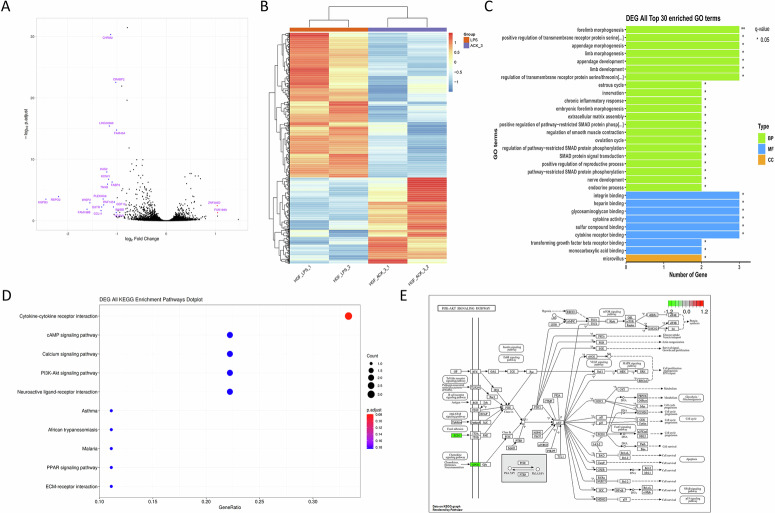
RNA-seq analysis indicated that the PI3K-Akt pathway is a potential target of Antcin K in HGFs. **A** The volcano plot shows the fold change in gene expression after Antcin K treatment. **B** The result of a heatmap of RNA sequencing showing differentially expressed genes in HGFs cells with or without Antcin K treatment. **C** The biological process and cellular function are analysed by the GO database. **D** Enrichment signaling pathways are analysed by the KEGG database. **E** Enrichment figure presenting pathways that were involved in the PI3K-Akt pathway.

**Fig. 3:**
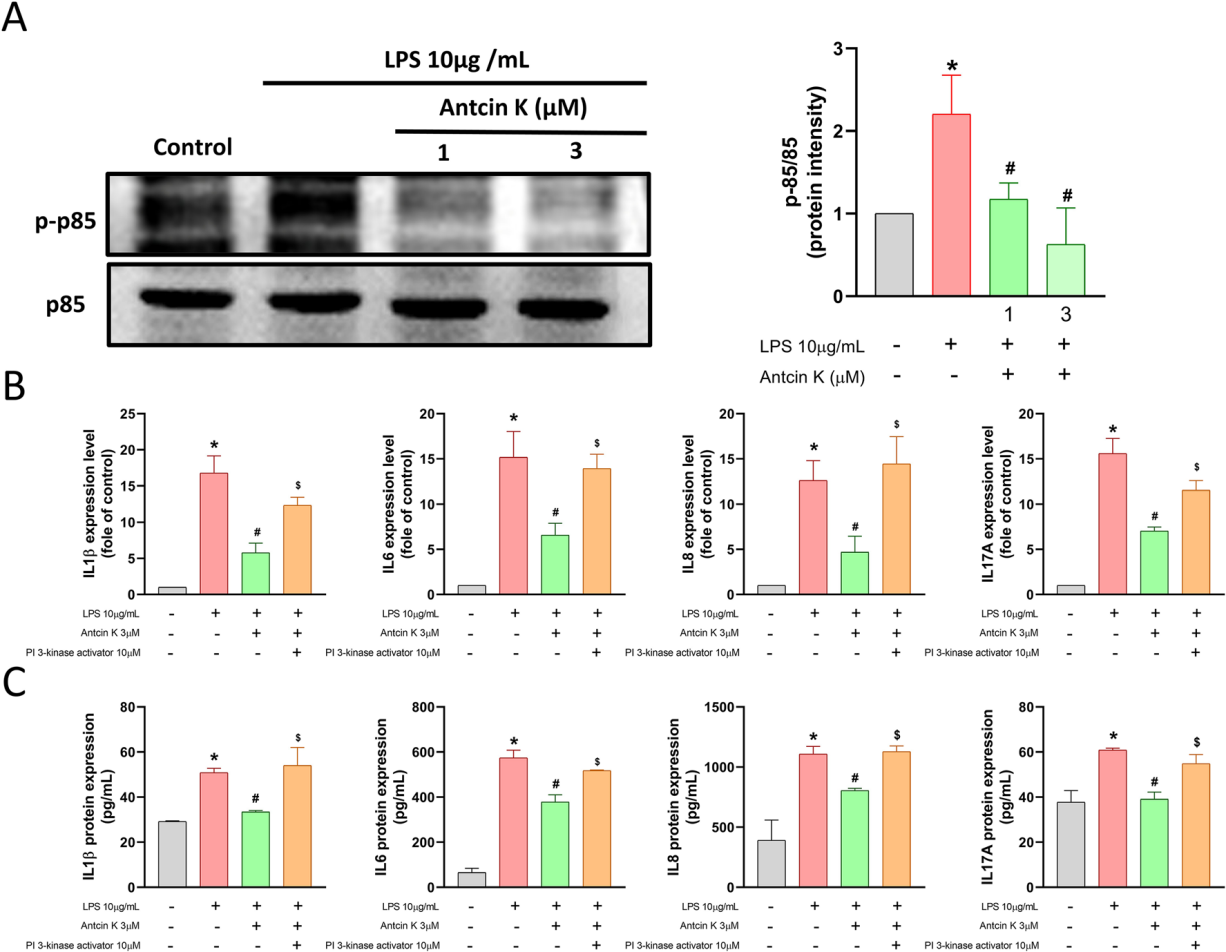
Antcin K blocks proinflammatory cytokines generation in HGFs via PI3K pathway. **A** HGFs were stimulated with LPS plus Antcin K, the p-p85 and p85 expression was performed by Western blotting. **B**, **C** HGFs were stimulated with PI3K activator (10 μM) and then applied with LPS and Antcin K for 24 h, the mRNAs and protein expression was analysed by qPCR and ELISA assay. *p < 0.05 compared with the control group. ^#^*p* < 0.05 compared with the LPS-stimulated group. ^$^*p* < 0.05 compared with the Antcin K-stimulated group.

**Fig. 4:**
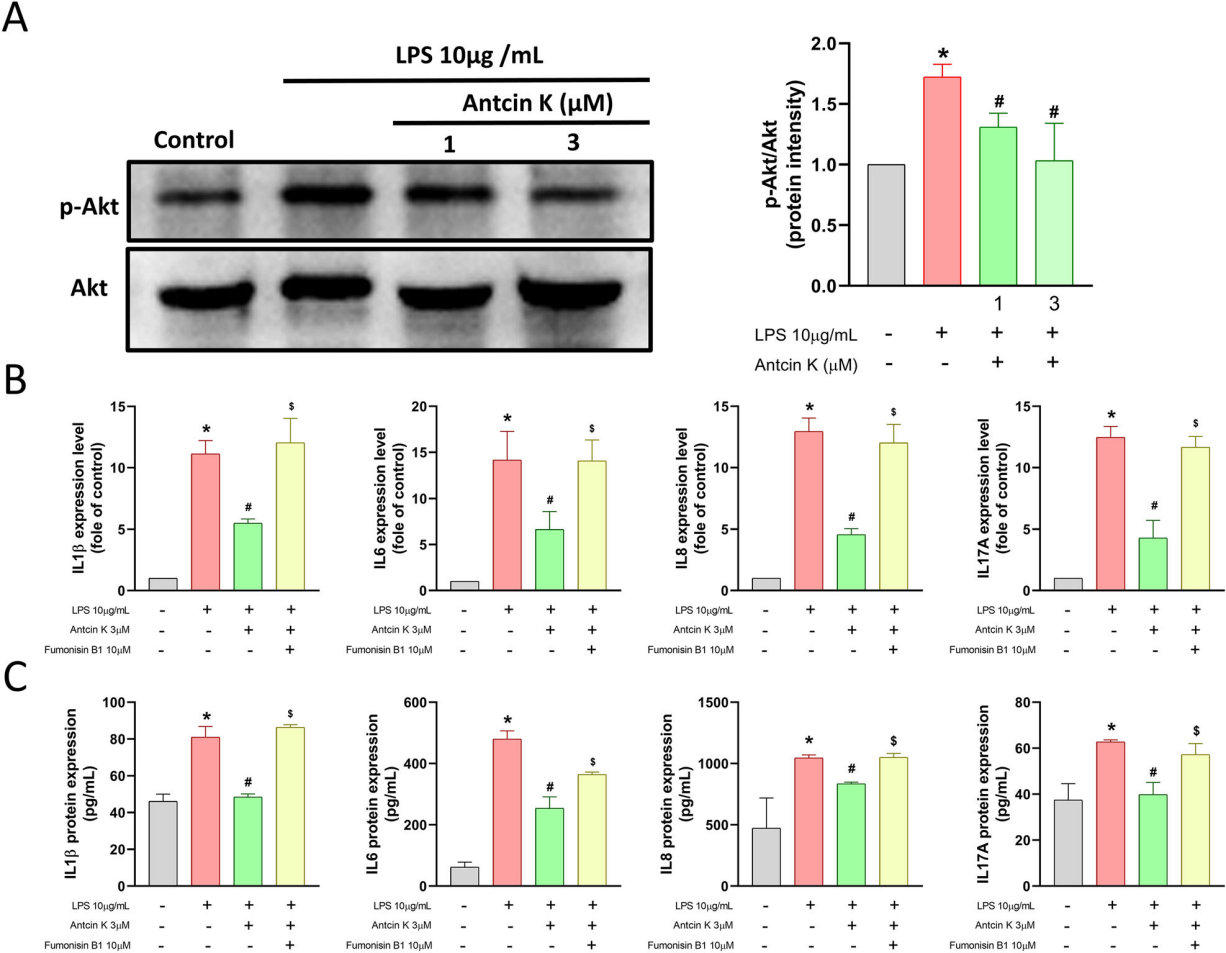
Antcin K reduces proinflammatory cytokines production in HGFs through Akt pathway. **A** HGFs were stimulated with LPS plus Antcin K, the p-Akt and Akt expression was performed by Western blotting. **B**, **C** HGFs were stimulated with Akt activator (Fumonisin B1; 10 μM) and then applied with LPS and Antcin K for 24 h, the mRNAs and protein expression was analysed by qPCR and ELISA assay. *p < 0.05 compared with the control group. ^#^*p* < 0.05 compared with the LPS-stimulated group. ^$^*p* < 0.05 compared with the Antcin K-stimulated group.

**Fig. 5:**
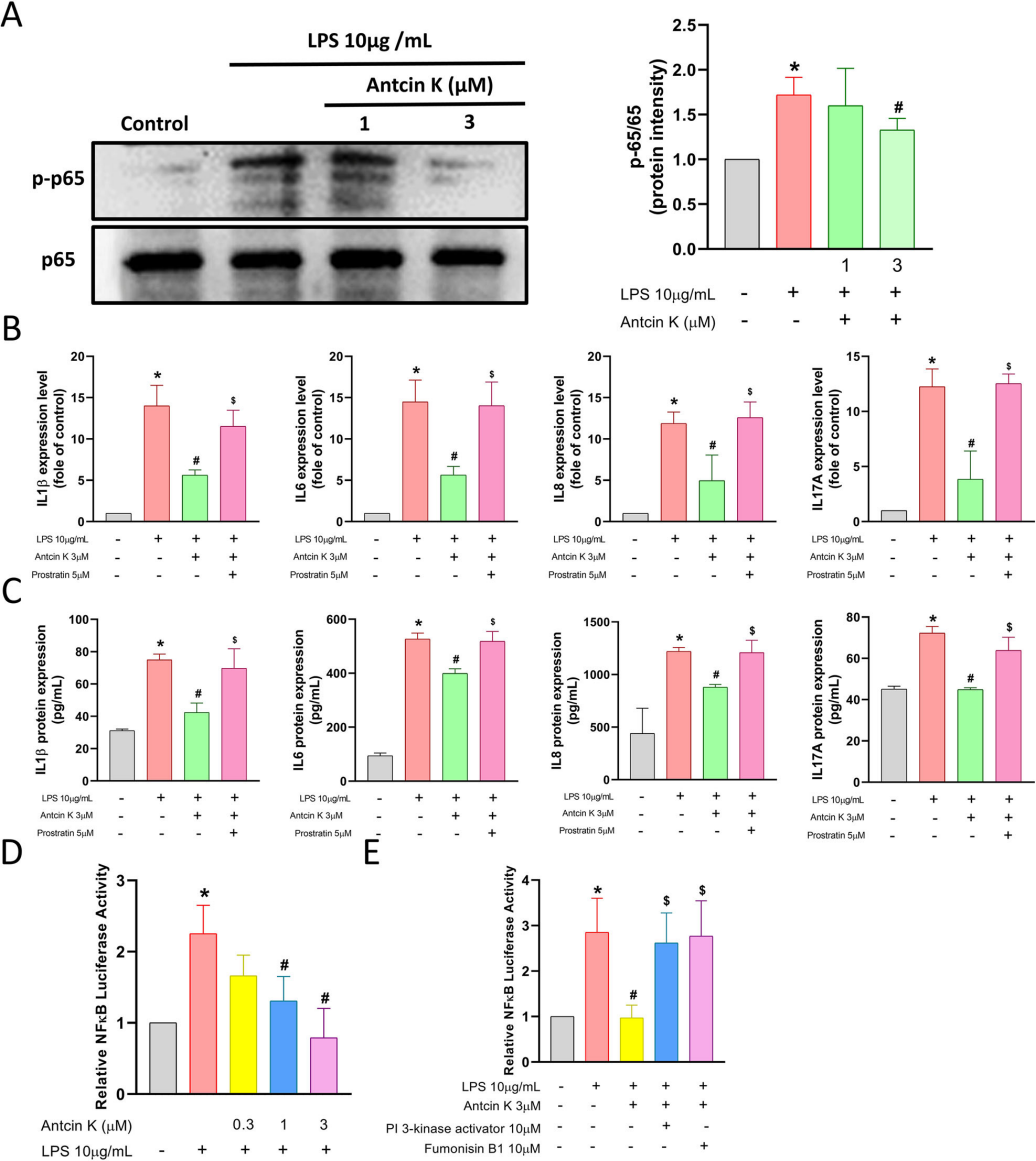
NF-κB pathway is regulated Antcin K-inhibited proinflammatory cytokines production in HGFs. **A** HGFs were stimulated with LPS plus Antcin K, the p-p65 and p65 expression was performed by Western blotting. **B**, **C** HGFs were stimulated with NF-κB activator (Prostratin; 5 μM) and then applied with LPS and Antcin K for 24 h, the mRNAs and protein expression was analysed by qPCR and ELISA assay. (**D** & **E**) HGFs were stimulated with pharmacological activators and then applied with LPS and Antcin K for 24 h, the NF-κB luciferase activity was performed. *p < 0.05 compared with the control group. ^#^*p* < 0.05 compared with the LPS-stimulated group. ^$^*p* < 0.05 compared with the Antcin K -stimulated group.

To explore the molecular mechanisms responsible for the anti-inflammatory effects of Antcin K, we explored RNA-seq analysis on HGFs treated with or without Antcin K. Volcano and heatmap plots revealed various gene expression changes after Antcin K treatment (Fig. 2A, B). Gene Ontology (GO) biological process analysis suggested that chronic inflammatory response and cytokine activity were involved, which are associated with inflammatory disorders (Fig. 2C). KEGG enrichment pathway analysis revealed that the PI3K-Akt signaling pathway, including PI3K, Akt, and NF-κB mechanisms, was mediated by Antcin K (Fig. 2D, E). Treatment of HGFs with Antcin K inhibited LPS-induced PI3K and Akt phosphorylation (Figs. 3A and 4A). The PI3K activator and Akt activator (Fumonisin B1) antagonized Antcin K-regulated reduction of proinflammatory cytokines production in HGFs (Figs. 3B, C and 4B, C). Furthermore, Antcin K also blocked LPS-induced phosphorylation of p65 (Fig. 5A). Incubation with the NF-κB activator (Prostratin) reversed the effects mediated by Antcin K (Fig. 5B, C). Additionally, Antcin K reduced NF-κB luciferase activity, which was restored by PI3K and Akt activation (Fig. 5D, E). Thus, Antcin K mitigates proinflammatory cytokine generation in HGFs via the PI3K, Akt, and NF-κB pathways.

### Antcin K reduces ligature-augmented inflammatory cytokines production and periodontitis in vivo

**Fig. 6:**
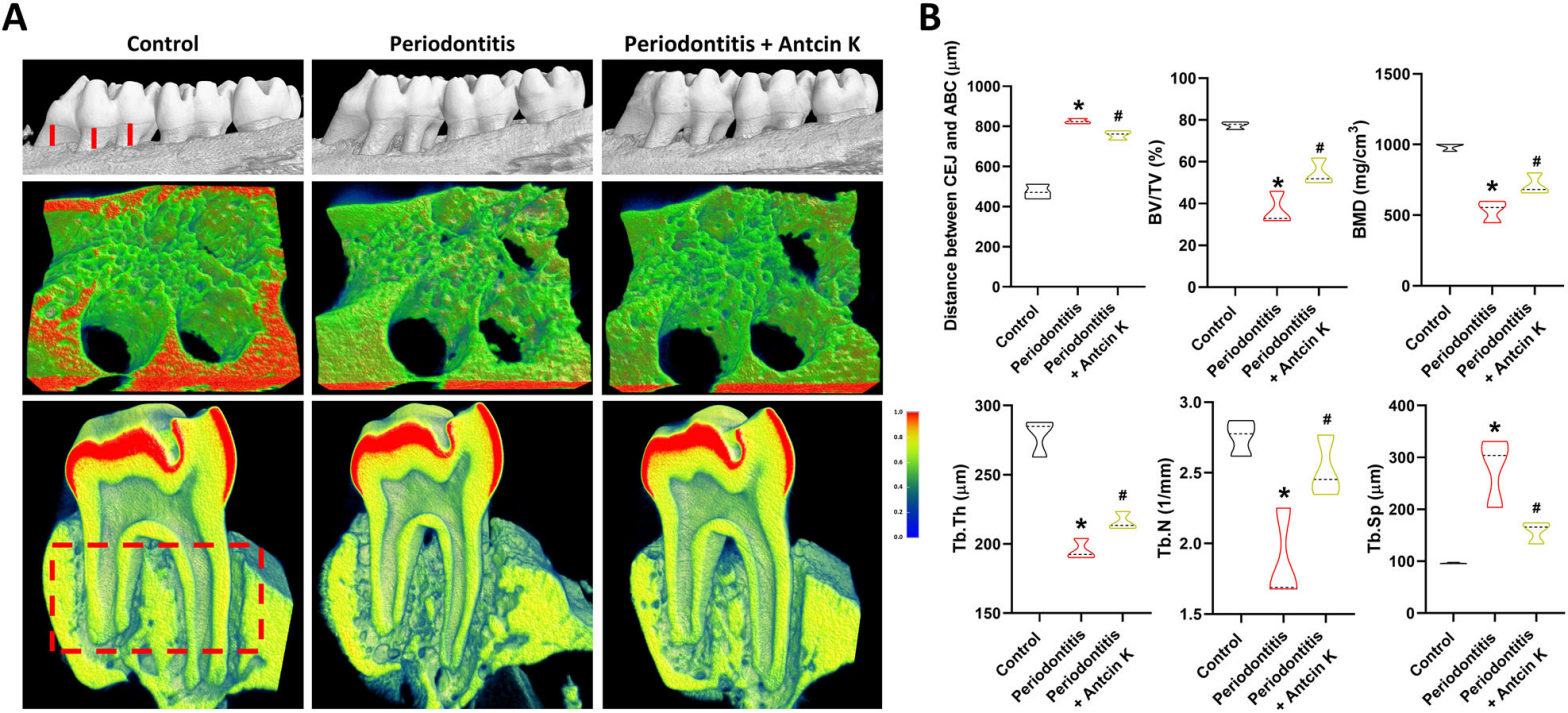
Antcin K blocks ligature-promoted periodontitis in vivo. **A** μCT images from left side of the maxilla of control, ligature and ligature+Antcin K groups. **B** Quantitative results of the CEJ-ABC distances, BV/TV, BMD, Tb.Th, Tb.N and Tb.Sp. *p < 0.05 compared with the control group. ^#^*p* < 0.05 compared with the PD group.

**Fig. 7:**
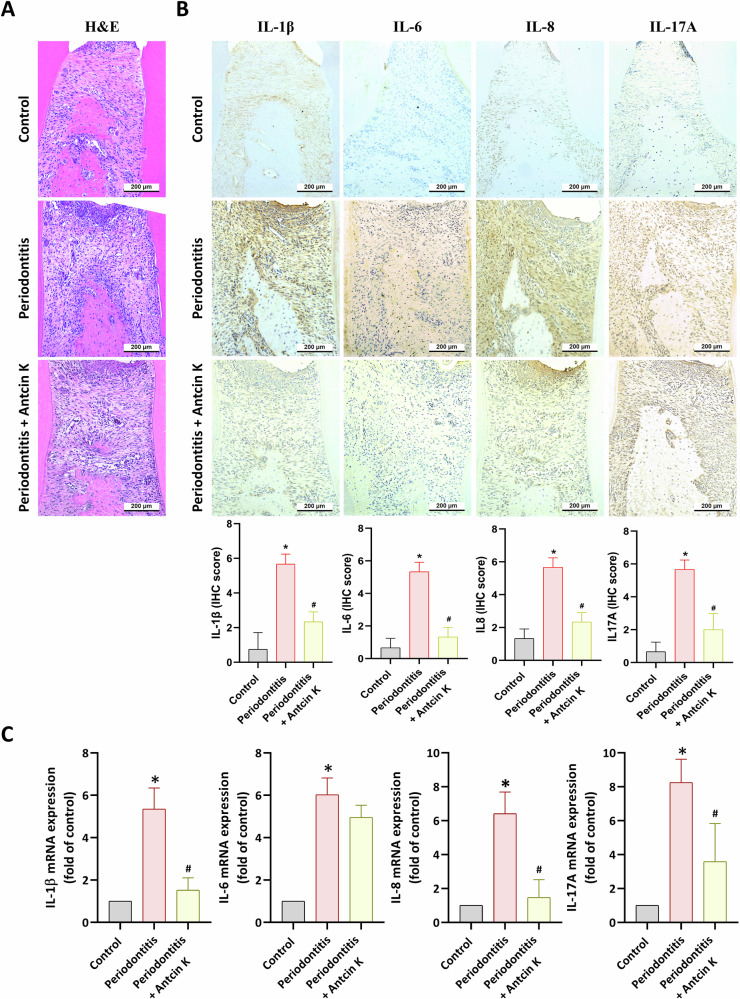
Antcin K blocks proinflammatory cytokines generation in vivo. **A** H&E staining and **B**, **C** IHC and qPCR analysis of IL-1β, IL-6, IL-8 and IL-17A in maxilla. *p < 0.05 compared with the control group. Scale bar = 200 μm. ^#^*p* < 0.05 compared with the PD group.

Next, we performed a ligature-induced periodontitis study to investigate the inhibitory roles of Antcin K in vivo. The ligature group exhibited an increased cemento-enamel junction (CEJ) to alveolar bone crest (ABC) distance, as shown by μCT results. In contrast, the Antcin K-treated group displayed inhibitory effects on bone loss (Fig. 6A, B). Additionally, Antcin K reversed ligature-induced reductions in bone volume/total volume (BV/TV), bone mineral density (BMD), trabecular thickness (Tb.Th), and trabecular number (Tb.N), while preventing the increase in trabecular separation (Tb.Sp) (Fig. 6B). Furthermore, H&E staining demonstrated that Antcin K treatment reversed ligature-augmented thinning and discontinuity of the junctional epithelium (Fig. 7A). Interestingly, qPCR analysis and IHC staining revealed that the Antcin K-treated group exhibited lower levels of IL-1β, IL-6, IL-8, and IL-17A in the periodontal tissues compared to the ligature group (Fig. 7B, C).

## Discussion

Numerous inflammatory cytokines play a critical role in the pathogenesis of periodontitis by interacting with immune and tissue cells [23]. The balance of these cytokines, regulated by immune responses, significantly influences the progression of inflammation [24]. Several studies have shown that levels of IL-1β, IL-6, IL-8, and IL-17A are elevated in periodontal tissues of patients with periodontitis compared to healthy controls [25, 26], driving disease progression. In this study, we found that Antcin K significantly reduces LPS-induced proinflammatory cytokine production in HGFs.

HGFs are vital connective tissue cells that play a key role in tissue healing and maintaining integrity [27]. Recently, they have also been recognized as critical immune system sentinel cells [28], actively modulating inflammation through cytokine synthesis and shaping tissue microenvironments [29]. Given their role in periodontal disease, HGFs have been established as a model for studying the inflammatory response in this context [30]. In our study, we demonstrated that LPS administration to HGFs triggers proinflammatory cytokine production, which is antagonized by Antcin K, inhibiting the synthesis of IL-1β, IL-6, IL-8, and IL-17A. Antcin K treatment also antagonized LPS-facilitated mRNA and protein synthesis of these cytokines, implying that Antcin K inhibits periodontal disease progression due to its strong anti-inflammatory activity. These findings highlight Antcin K’s potent anti-inflammatory activity and its potential to mitigate periodontal disease progression.

Ligation-induced experimental periodontitis is a well-established model in periodontology [31], wherein cervical ligation leads to plaque accumulation and sulcular epithelial ulceration [32]. This procedure activates the host immune response, resulting in bone resorption and infiltration of inflammatory cells into the gingival tissues. Importantly, Antcin K treatment also inhibited ligature-promoted periodontitis in vivo. Therefore, we suggest that Antcin K could be a potential therapeutic candidate for treating periodontal disease.

Investigating potential molecular mechanisms is a critical process in drug discovery. In this study, Antcin K-treated HGFs utilizing RNA-seq, the PI3K-Akt signaling, which includes PI3K, Akt, and NF-κB, is a prime candidate signaling pathway. PI3K-Akt signaling cascades play a critical function in different cellular processes, for instance migration, inflammation, differentiation and apoptosis [33, 34]. Antcin K stimulation reduces LPS-induced PI3K and Akt activation. The activators of PI3K and Akt antagonized Antcin K-mediated inhibition of proinflammatory cytokines synthesis. NF-κB is a key transcription factor downstream of the PI3K/Akt pathway, regulating inflammatory cytokine production [33]. Notably, Antcin K treatment inhibited p65 phosphorylation. Additionally, the NF-κB activator reversed the inhibitory performance of Antcin K. The suppression of LPS-enhanced NF-κB luciferase activity by Antcin K was restored by PI3K and Akt activators, indicating that the PI3K, Akt, and NF-κB pathways regulate Antcin K’s anti-inflammatory effects. We used RNA-seq and pharmacological activators to investigate the effects of the PI3K, Akt, and NF-κB pathways in Antcin K’s functions. However, a limitation is the lack of genetic activator materials to confirm these effects. Future studies could employ genetic activators of the PI3K, Akt, and NF-κB pathways to validate the effects of Antcin K on these pathways.

**Fig. 8:**
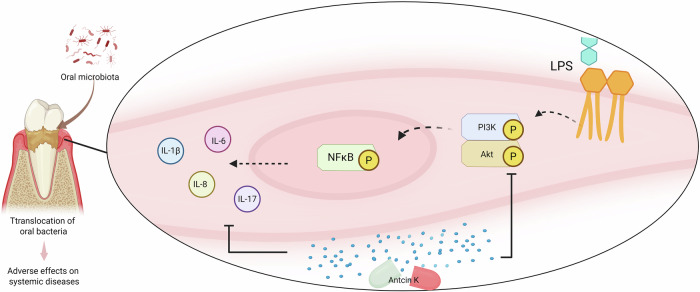
Illustration depicting the therapeutic effects of Antcin K in periodontal disease. Antcin K inhibits LPS-induced proinflammatory cytokines production in HGFs. The PI3K, Akt, and NF-κB pathways mediate Antcin K’s inhibitory effects. The in vivo study demonstrated that Antcin K blocks periodontal disease development in a ligature-mediated periodontitis.

Pharmacotherapy has magnificently availed by the identification of natural substances and their structural analogs [35, 36]. Taiwanese traditional medicine has utilized the unusual fungus *A. cinnamonomea* for millennia to remedy inflammatory disorders, hypertension, liver illnesses, and tumor [37]. It has been reported that Antcin K possesses anti-inflammatory abilities that markedly lower the synthesis of IL-6, IL-1β, and TNF-α [38]. The anti-inflammatory action in arthritic disorder is regulated by Antcin K, a functional molecule that is isolated from the fruiting bodies of *A. cinnamomea* [39]. In this study, we revealed a novel therapeutic function of Antcin K, which inhibits LPS-induced proinflammatory cytokine production in HGFs. The PI3K, Akt, and NF-κB pathways mediate Antcin K’s inhibitory effects. Additionally, our in vivo study demonstrated that Antcin K blocks periodontal disorder development in a ligature-mediated periodontitis model (Fig. 8).

In conclusion, Antcin K demonstrates significant potential as a therapeutic agent for periodontal disease. By inhibiting key inflammatory cytokines and modulating the PI3K, Akt, and NF-κB pathways, Antcin K reduces inflammation and prevents the progression of periodontitis. Given its natural origin and anti-inflammatory properties, Antcin K represents a promising candidate for further clinical studies in the treatment of periodontitis.

## Material and methods

The antibodies against p-p85 (4228S, 1:1000), p85 (4257S, 1:1000) and p-p65 (3033, 1:1000) from Cell signaling (Danvers, MA, USA); p-Akt (sc-5298, 1:1000), Akt (sc-16646-R, 1:1000), p65 (sc-8008, 1:1000) as well as PI3K activator (sc-3036), Akt activator (Fumonisin B1, sc-201395) and NF-κB activator (Prostratin, CAS60857-08-1) from Santa Cruz (Dallas, TX, USA). Cell culture supplements and Lipofectamine 2000 were bought from Invitrogen (Carlsbad, California, USA). All additional reagents and *Pg* LPS were supplied by Sigma-Aldrich (St. Louis, Missouri, USA).

### Cell culture

Human gingival connective tissues that were discarded were used to make the HGFs. The China Medical University Hospital’s Institutional Review Board gave its approval to the study protocol (CMUH112-REC3-188). Following a previously published protocol [40], the HGFs were cultivated in RPMI 1640 medium supplemented with 10% decomplemented fetal bovine serum (HyClone, Logan, UT, USA), 100 U/mL penicillin, and 100 mg/mL streptomycin.

### MTT assay

The 96-well culture plates were filled with HGFs and treated with or without different amounts of Antcin K. Dimethylsulfoxide was used to dissolve the MTT buffer, which was added at a concentration of 0.5 mg/mL. An absorbance measurement at 570 nm was performed with a BioTek microplate reader (Winooski, Vermont, USA) [18, 41].

### RNA sequencing (RNA-Seq) and data analysis

Total RNA was isolated from the HGFs treated with or without Antcin K using TRIzol reagent. RNA sequencing was performed by Biotools (New Taipei City, Taiwan) on an Novaseq X Platform. Data were analysed using the Biotools Cloud Platform (https://cloud.toolsbiotech.com/login) [42]. Differentially expressed genes (DEGs) identified from RNA-seq analysis were submitted to the Kyoto Encyclopedia of Genes and Genomes (KEGG; genome.jp/kegg/pathway.html) database to explore potential pathways and analyze biological functions.

### Real-time quantitative polymerase chain reaction amplification

As directed by the manufacturer, RNA was isolated from HGFs using a TRIzol kit (MDBio, Taiwan). Using a NanovueTM Spectrophotometer (GE Healthcare, WI, USA), the quantity and quality of the RNA were evaluated based on A260 readings. cDNA synthesis was performed using an M-MLV RT kit (Invitrogen, CA, USA) and 1 μg of total RNA in accordance with the manufacturer’s instructions. The KAPA SYBR® FAST qPCR Kit was supplied by Applied Biosystems (CA, USA), to perform real-time quantitative polymerase chain reaction (qPCR) [18, 41].

### Western blot analysis

The extracted proteins (30 μg) were resolved using SDS-PAGE, and PVDF membranes were then transferred in accordance with the protocols described in our previous publications [43, 44]. After blocking the membranes for an hour in PBST containing 4% non-fat milk, they were treated with primary antibodies and secondary antibodies conjugated with HRP for an additional hour. The Fujifilm LAS-3000 imaging equipment was used to view the blot membranes. A computer densitometer equipped with an ImageQuant LAS4000 (GE Healthcare Life Sciences) was used to measure the amount of protein.

### ELISA assay

The amount of IL-1β (DY201-05), IL-6 (DY206), IL-8 (DY208) and IL-17 (DY317) expressed in the cell culture media was assessed using an ELISA kit from R&D Systems (Minneapolis, MN, USA). As directed by the manufacturer, the cell culture media were collected and subjected to ELISA analysis for IL-1β, IL-6, IL-8 and IL-17 production after a 24-h Antcin K treatment of HGFs [45, 46].

### NF-κB luciferase activity

The NF-κB luciferase plasmid (Stratagene; MO, USA) was transfected into HGFs using Lipofectamine 2000 (Invitrogen; Carlsbad, CA, USA). After that, pharmacological activator and Antcin K were applied for a full day. Luciferase activity was measured using the dual luciferase assay equipment in compliance with the manufacturer’s guidelines [47].

### Ligature-induced periodontitis model

The National Laboratory Animal Center in Taipei, Taiwan, provided the eight-week-old Wistar rats for the experiment. The Animal Research Ethics Committee gave its approval to all animal treatments. Following a 1-week adaptation period with daily monitoring, rats were randomly assigned to three groups (n = 6 per group): Control, PD (Periodontitis), and Antcin K. Under anesthesia (zoletil 80 mg/kg and xylazine 10 mg/kg), experimental periodontitis was induced by placing 3–0 silk ligatures (UNIK, Taipei, Taiwan) around the bilateral maxillary first molars (M1), with the knot secured to the mesial surface using composite resin. Control group animals received no ligatures. The Antcin K group received intraperitoneal injections of Antcin K (20 mg/kg) every other day, while Control and PD groups received normal saline injections.

Seven days post-ligation, rats were sacrificed. The left M1 gingival tissues were collected and preserved in Allprotect Tissue Reagent (Qiagen, Valencia, CA) at -20°C. The maxilla was fixed in 4% paraformaldehyde for 24 h before transfer to 70% ethanol solution. The left maxilla specimens were analyzed using a Bruker SkyScan 2211 micro-CT system (Kontich, Belgium) under previously established parameters [48, 49].

### Histopathological evaluation

The right maxilla underwent decalcification in 10% EDTA solution for 4 weeks at 4°C, followed by paraffin embedding and preparation of 5-μm bucco-lingual sections for hematoxylin and eosin (H&E) staining. For immunohistochemistry (IHC) staining, the tissues were treated with primary antibodies. They used a Leica Novolink Polymer Detection system (Leica Biosystems Inc., IL, US) for DAB staining and secondary antibody binding. Using techniques already outlined in our prior research, the staining data were quantified [50]. IHC analyses were performed (n = 3 mice per group) using the following primary antibodies: IL-1β (1:500; Novus Biologicals; 2805), IL-6 (1:500; Abcam; ab9324); IL-8 (1:500, GeneTex; GTX115959), IL-17A (1:500, GeneTex; GTX17587).

### Statistical analysis

The statistical significance was assessed using the Student’s t-test, and the analysis of the control group and the experimental multiple groups was performed using a two-way ANOVA. Results are expressed as the mean ± standard deviation (SD). *p* < 0.05 is considered statistically significant.

## Supplementary information

Original image for checking - Western blot full gel
