# Supplementary figures and images for: Antcin K suppresses proinflammatory cytokines expression via the PI3K, Akt and NF-κB pathways in human gingival fibroblasts: implications for periodontitis treatment
Source: Cell Death Discov. 2025 Nov 22;12:25. doi: 10.1038/s41420-025-02865-3 (PMC12808328; doi:10.1038/s41420-025-02865-3)

Fig 3

HGF cell

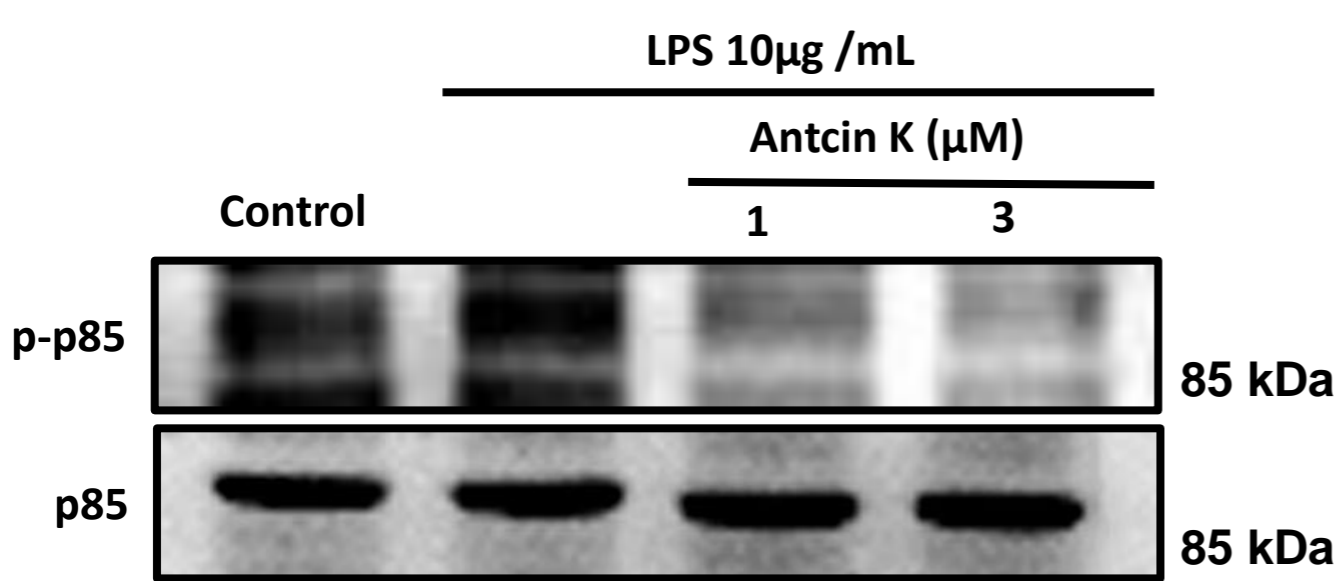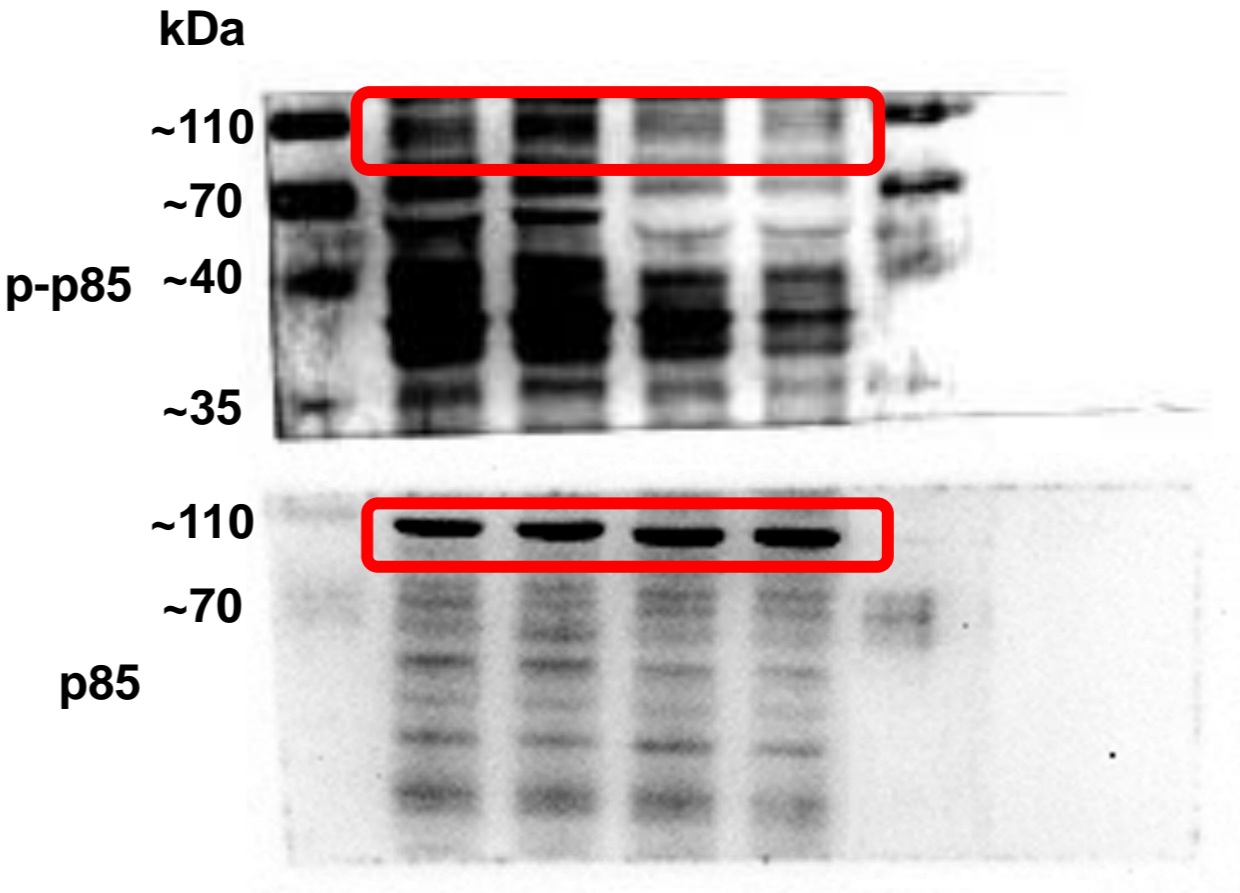

Fig 4

HGF cell

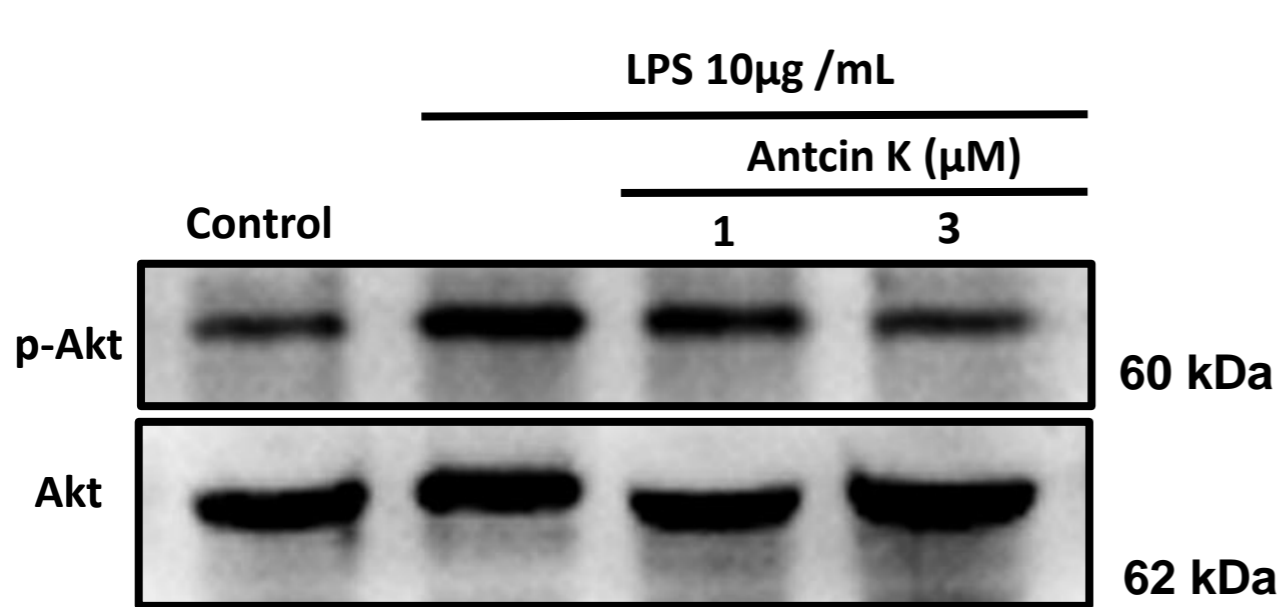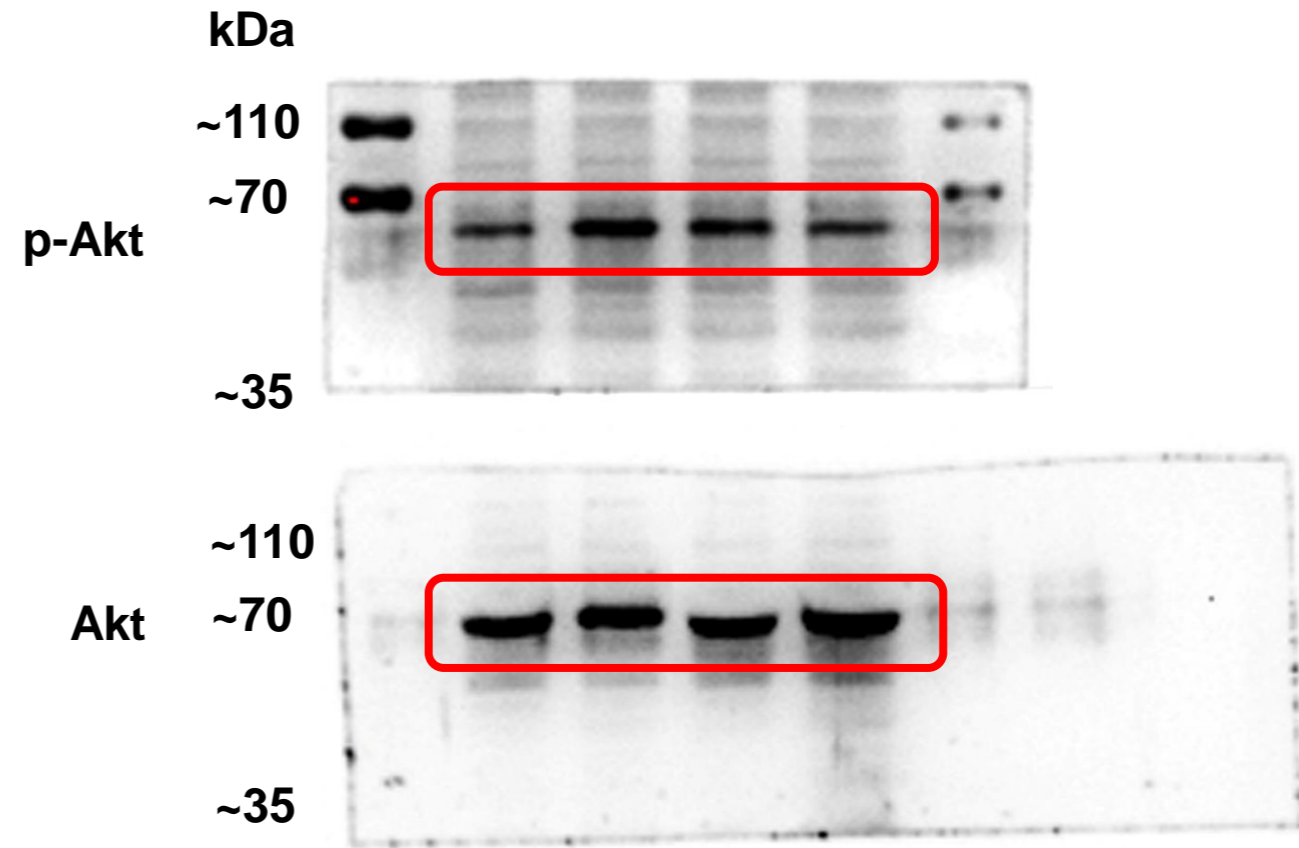

Fig 5

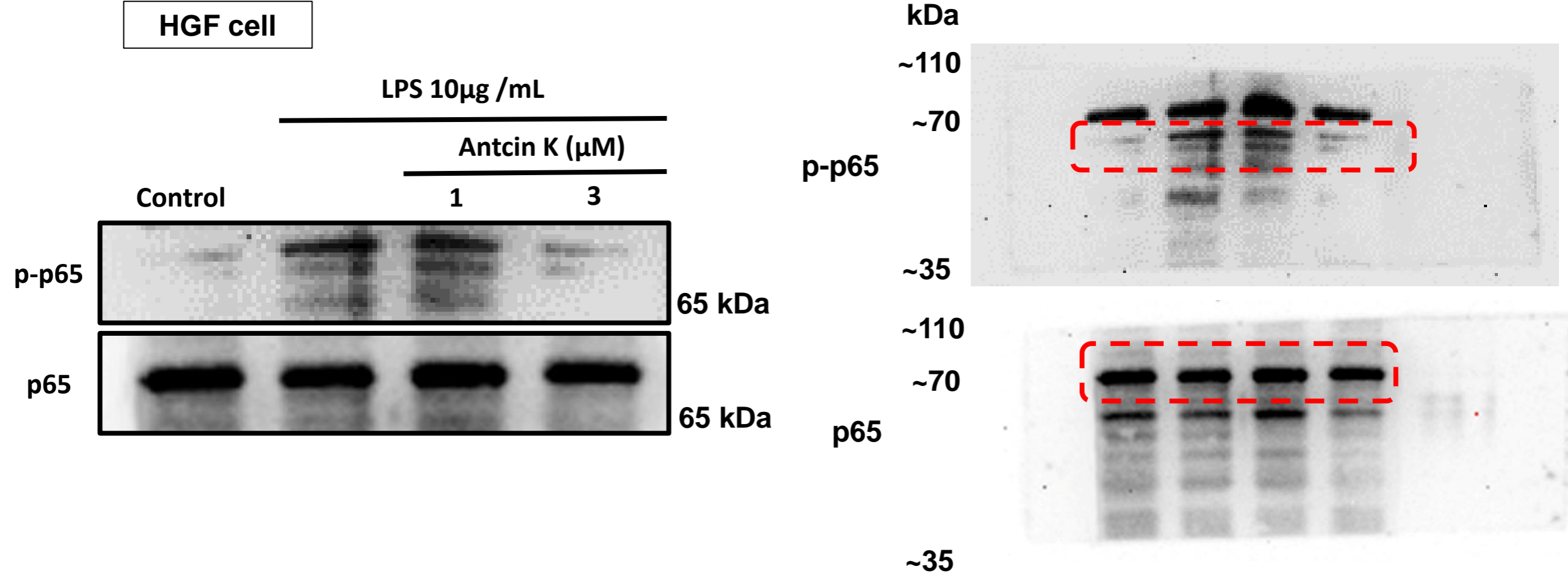

Supplement: Supplementary file 1 — Original image for checking - Western blot full gel [file 41420_2025_2865_MOESM1_ESM.pdf]
